# Extracting Social Information from Chemosensory Cues: Consideration of Several Scenarios and Their Functional Implications

**DOI:** 10.3389/fnins.2015.00439

**Published:** 2015-11-20

**Authors:** Yoram Ben-Shaul

**Affiliations:** Department of Medical Neurobiology, Hebrew University Medical SchoolJerusalem, Israel

**Keywords:** chemosensory cue, social communication, olfactory circuitry, traits, neuronal computation, pheromones

## Abstract

Across all sensory modalities, stimuli can vary along multiple dimensions. Efficient extraction of information requires sensitivity to those stimulus dimensions that provide behaviorally relevant information. To derive social information from chemosensory cues, sensory systems must embed information about the relationships between behaviorally relevant traits of individuals and the distributions of the chemical cues that are informative about these traits. In simple cases, the mere presence of one particular compound is sufficient to guide appropriate behavior. However, more generally, chemosensory information is conveyed via relative levels of multiple chemical cues, in non-trivial ways. The computations and networks needed to derive information from multi-molecule stimuli are distinct from those required by single molecule cues. Our current knowledge about how socially relevant information is encoded by chemical blends, and how it is extracted by chemosensory systems is very limited. This manuscript explores several scenarios and the neuronal computations required to identify them.

## Introduction

In many species, chemosensory cues are crucial for obtaining information about the environment and particularly for interactions with other individuals (Wyatt, 2014). At one end of the spectrum are tasks involving single compounds, such as detection of females by male moths (Sakurai et al., 2014). Here, a communication system involving emission of one specific compound has likely co-evolved with a dedicated sensory processing channel. A related situation in vision is phototaxis: attraction to high photon levels, also occurring in moths and other insects (Yamaguchi and Heisenberg, 2011). At the other extreme is a task like face recognition which requires a complex computation involving sampling and comparison of relational information across multiple detectors. This article is motivated by the view that analyses of chemosensory scenes involve non-trivial comparison of information across compounds and receptors, a task far more challenging than detection of levels of single-compounds.

The chemical profile associated with any animal, and mammals in particular, is a highly complex mix of various molecules that can convey information about the emitting organism (Albone and Shirley, 1984). One important distinction is between pheromones, which elicit some response but are anonymous with respect to the sender, and signature mixtures, which provide information about individuality or colony/family identity (Wyatt, 2010). In addition, social information can involve detection of specific *traits*. Throughout this manuscript, the term *trait* refers to a particular property of an individual, which may be relevant for guiding behavior toward it. A trait may be permanent (e.g., *species, sex*) or temporary (e.g., *age, health status*). Such information is conveyed by chemical cues that cannot be strictly defined as either pheromones or as signature mixes. Yet, they are clearly important for guiding behavior.

Sensory systems evolved to extract those statistical features that are relevant for identifying information important to the organism (Rieke et al., 1995; Barlow, 2001; Bradbury and Vehrencamp, 2011). Understanding a sensory system therefore requires identification of the problems that it must solve. For example, detection of light and identification of specific faces involve distinct statistical features and therefore, distinct neural networks (Purves, 2012). Likewise, chemosensation can serve in various contexts involving distinct computations. The problems solved by the olfactory system are varied and include stimulus identification (Chapuis and Wilson, 2012; Rokni et al., 2014), discrimination among stimuli (Kepecs et al., 2007), or source tracking (Thesen et al., 1993; Cardé and Willis, 2008). The chemosensory task considered here is the recognition of specific *traits* using chemical cues. Specifically, this manuscript focuses on the links between specific distributions of chemical cues and potential neuronal solutions to detect them. Our approach is to examine various scenarios of chemosensory cue distributions and to spell out the steps required to extract the relevant information under each of them. Evidence for some scenarios is well established, while others are more speculative. The hope is that an explicit description of the required computations, even if abstract, will help to eventually identify of the actual circuit elements that realize these computations.

## Methods

All plots illustrating specific networks and decision rules were created using MATLAB code. The purpose of the networks is to demonstrate the logical steps involved, and not to implement any realistic neuronal modeling. In other words, the purpose is to show *what* must be calculated, but not *how*. Therefore, the code realizes the networks in a very literal manner. For example, linear input units such as appear in many of the networks (e.g., **Figure 2A**) were implemented by the following code: *R* = *max*(0,(*C-T*)·*G*), where *R* is the response of a unit, with a threshold *T*, and a gain *G*, to a stimulus concentration *C* (Supplementary code file: thresh_unit). The *max* condition ensures that a response will only occur if the concentration *C* is larger than the threshold, *T*. The output unit in **Figure 2A** was realized by subtracting the responses of two such linear input units with different gains and thresholds and applying a threshold to the (normalized) response (Supplementary code file: soft_range_unit). The plots in **Figures 2B,C** were created using the script simulate_one_compound_scenarios which itself calls the soft_range_unit function with a set of concentrations that was randomly sampled from a uniform random distribution in the range [0, 10]. All manuscript figures showing outputs of other networks were generated in an analogous manner using specific MATLAB scripts. The code is extensively documented to explain each of the calculations and a *readme* file lists which scripts were used for each figure (Supplementary Material)

## Results

**Figure 1:**
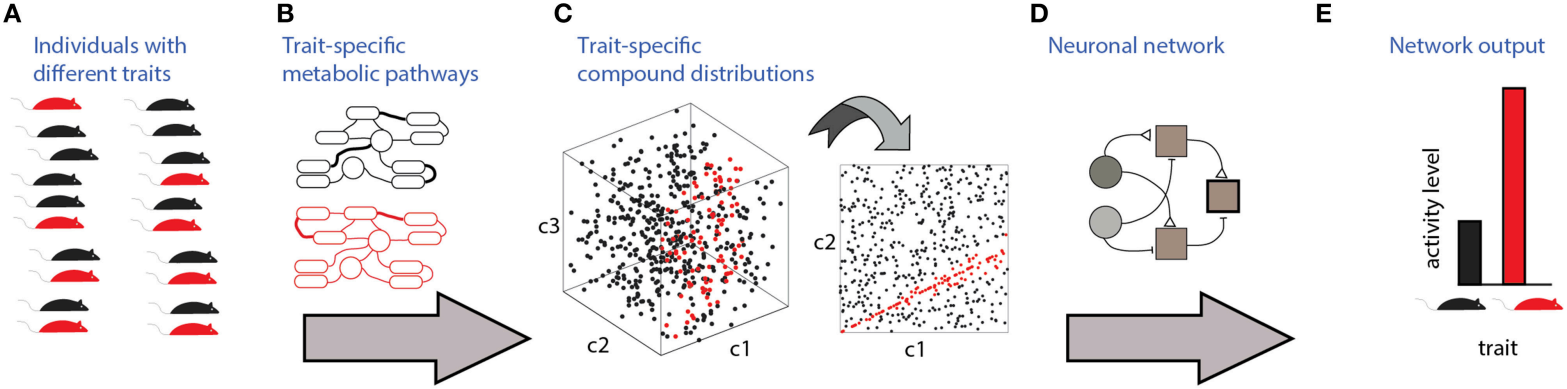
**Stages involved in obtaining social information from chemical cues**. Different traits (red or black in **A**) are associated with distinct metabolic pathways/expression patterns **(B)**, which lead to distinct profiles of emitted signals **(C)**. In this example, chemical space is defined by levels of 3 components, c1, c2, and c3. Distinguishing among the traits requires neuronal networks **(D)** that can identify the relevant features in chemical space. Here, the feature is a specific ratio between levels of compounds c2 and c1. Ultimately, these networks elicit neuronal activity **(E)** that can distinguish among chemosensory profiles associated with different traits.

Communication and detection of social information via chemosensation involves several stages which are illustrated in Figure 1. The source of the signals are individuals with specific *traits* (Figure 1A). To be detectable, these traits must be associated with particular distributions of molecules, which are determined by trait-specific metabolic pathways or gene expression patterns (Figures 1B,C). Finally, specific neuronal networks (Figure 1D) detect particular traits from profiles of chemosensory cues. Figure 1C depicts a three dimensional space (i.e., defined by three compounds), of which only two are relevant for detection (Figure 1C, right). In this hypothetical example, the relevant parameter is a specific *ratio* between the levels of two compounds, c1 and c2. The nervous system must therefore elicit a well-defined change in neuronal activity (Figure 1E) following the detection of a particular ratio. For simplicity, the traits considered here are binary, assuming one of two values. The networks are composed of units responding to excitatory or inhibitory inputs. The input units represent chemically selective receptor neurons, and thus respond monotonically, though not necessarily linearly, to concentrations of individual molecules. Each network includes one output unit, whose activity reflects the network's decision regarding the trait's value. The proposed networks are *not* intended to be biophysically realistic and are agnostic about particular neuronal codes (e.g., rate vs. temporal coding) and about the actual neuronal hardware used to implement the computations (e.g., dendritic vs. somatic integration). Instead, they serve to illustrate the essential computations required for each type of classification. In the Discussion, an attempt is made to map these model networks to specific elements of the olfactory system.

### Single compound codes and their limitations

**Figure 2:**
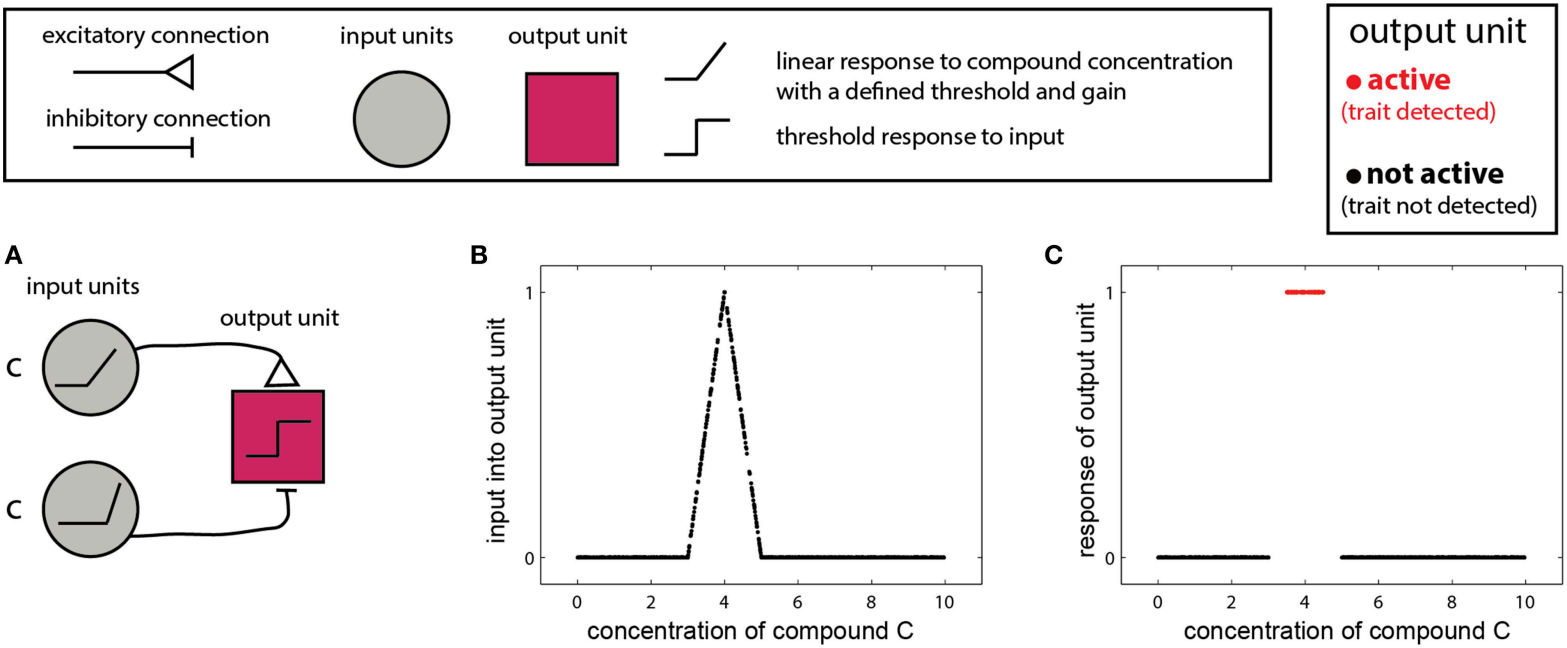
**Detection of a single compound**. **(A)** Simple network that detects the presence of a compound c in a specified range. The network comprises two linearly responding units. Note that the threshold of the inhibitory unit is higher and its response function is steeper (higher gain). A threshold response to the difference between the inputs from the two first-layer units results in a selective response in the output unit (red) only when c is within a specific level range. The range can be set by altering the response functions of the first-layer units and by the threshold of the output unit. **(B)** Input to the network in **(A)** as a function of the level of c. **(C)** Output of the network. Legend at top explains network elements.

In the simplest scenario, a trait is reflected by the presence or absence of one molecule. In this case, the detecting organism needs to detect whether the concentration of the molecule is above a certain threshold or within a given range. The single-compound scenario is related to the classic definition of a pheromone—namely, that the mere presence of one specific compound can elicit a particular behavioral outcome (Wyatt, 2010). Examples include the male silkworm moth's (*Bombyx mori*) response to bombykol (Butenandt et al., 1961), or suppression of mating in mice, due to detection of a peptide indicative of a juvenile state (Ferrero et al., 2013). In such cases, the stimulus space is one dimensional, and the corresponding networks are simple. One version involves two units: one excitatory and one inhibitory both of which respond linearly to the stimulus, above some threshold (Figure 2A). The excitatory unit has a lower threshold and gain, and is thus active at lower concentrations, while at higher concentrations the inhibitory unit is recruited, suppressing the output unit. The output unit implements a threshold on the integrated inputs (Figure 2B), so that its output represents a binary decision of whether the compound is, or is not, within a certain range (Figure 2C). Removal of the threshold operation in the last unit will lead to a continuous measure of similarity to some optimal cue level, as shown in Figure 2B. The slope of the response function in Figure 2B is a direct function of the slopes of the input units.

Taken to the extreme, under the one compound scenario, each biologically relevant trait is independently represented by levels of one specific compound. For example, levels of one compound would convey an individual's sex, another its age, a third its reproductive status and so on. While such a scheme would simplify *decoding*, it presents a very inefficient code since it requires dedicated metabolic pathways to generate compounds for each trait. Unique single-compound signatures of individuality, are particularly unfeasible. Another critical shortcoming of single compound codes is the likelihood of not being specific for a given species. For species that do not interact, this does not present a problem (Kelly, 1996). However, in some cases, the simplicity of the code facilitates mimicry and therefore allows a predatory species to bait a prey organism (Gemeno et al., 2000). An even more fundamental problem with single compound codes is their failure to provide invariant information. For example, if a particular trait is associated with a certain level of some compound, stimulus source dilution would present on obvious confounding factor. Likewise, vital information about the state of metabolic pathways is often manifest by the *relative* levels of several compounds, rather than absolute levels of individual compounds.

### Multi compound codes

Transmission of chemical information by combinations of multiple compounds is widespread (Wyatt, 2014). In the nematode *C. elegans*., different combinations of modular components can promote avoidance, reproduction, long range attraction, and developmental diapause (Srinivasan et al., 2012). Likewise, even though a single component may suffice to elicit a behavioral response, female moth signals are much more effective when present as a combination of components (Linn et al., 1987), thus providing specificity and minimizing interference among related moth species (Linn et al., 1988). A similar mechanism for species-specific mating, using multicomponent blends, is present in goldfish (Levesque et al., 2011). In social insects such as bees, communication is also based on complex codes involving combinations of multiple components (Slessor et al., 2005). Similarly, naked mole-rats, which are social mammals, use a unique odor signature, composed of multiple components, to identity colony members (Oriain and Jarvis, 1997). In mice, some volatiles act synergistically (Novotny et al., 1985) to elicit aggression, while particular combinations of major urinary proteins convey information about individuality (Cheetham et al., 2007; Kaur et al., 2014).

**Figure 3:**
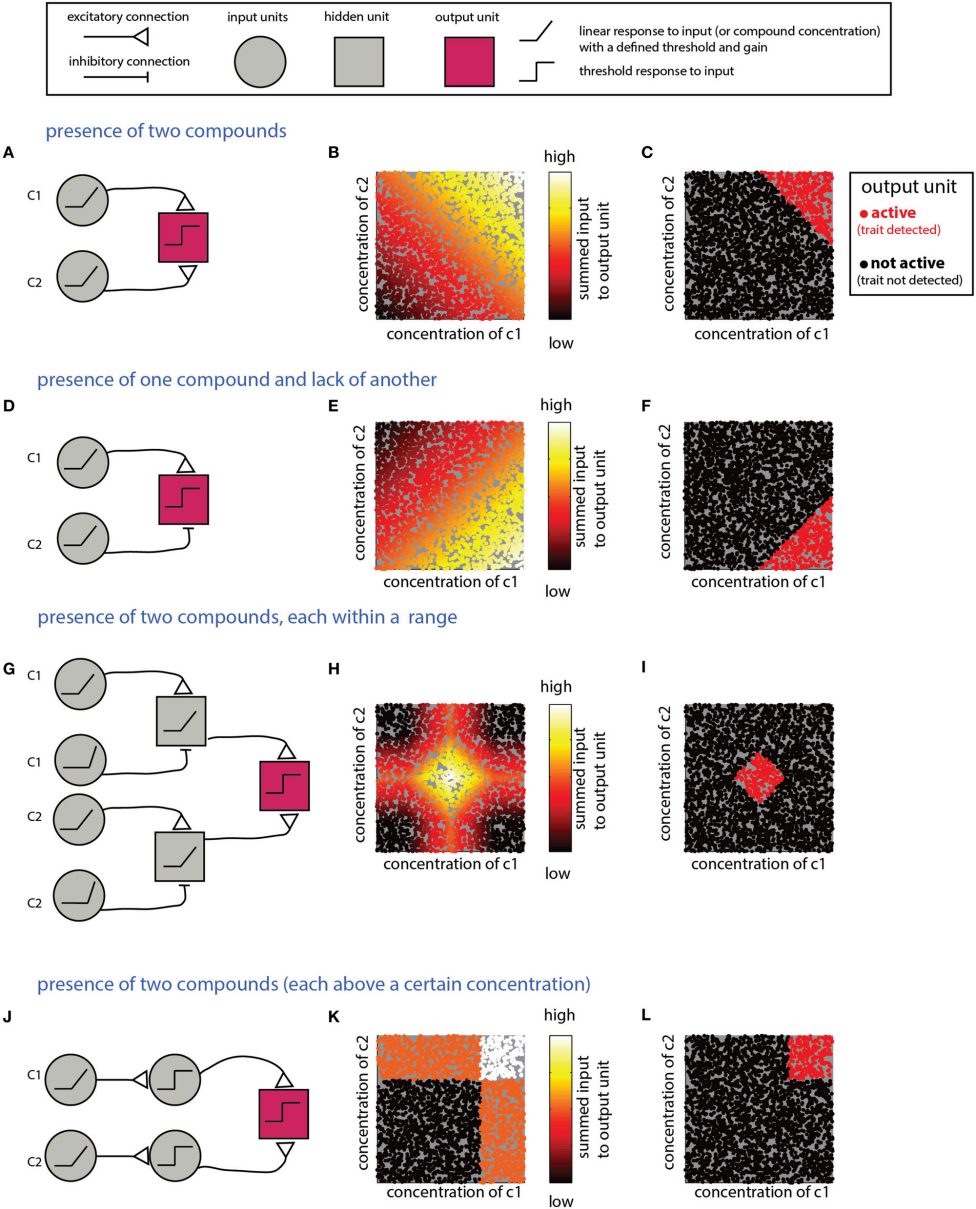
**Detection of multiple (two) compounds. (A–C)** Detection of the sum of two compounds. **(A)** A simple network that detects the presence of two compounds c1 and c2. The network comprises two linearly responding units that feed into a third integrating unit. **(B)** Inputs into the output unit in the network in **(A)** as a function of the levels of c1 and c2. The magnitude of the inputs is indicated by the color of the dots (arbitrary units). **(C)** Activity of the output unit after thresholding the input shown in **(B)**. **(D–F)** Detection of a difference between two compounds. Here, the output unit receives an excitatory input from a unit that detects c1 and an inhibitory input from a unit that detects c2. Assuming that the input units' response functions and efferent connections are similar, the output unit be active when c1 is larger than c2. **(G–I)** Detection that each of two compounds is within a specific range. **(G)** The output unit in this network receives inputs from two range-detecting units as shown in Figure 2A (with each network sensitive to the range of one of the compounds). **(H)** Input to the output unit in **(G)**. **(I)** Output of the output unit in **(G)** after thresholding. **(J–L)** A network that detects that *each* of two compounds is above some threshold. Similar modifications can be applied to all the other networks in this section, to impose conditions on each of the compounds. Legend at top explains network elements.

What mechanism is suitable for detecting a combination of individual compounds? An obvious solution involves summation of inputs from multiple neurons, each of which is responsive to one component. Figure 3A shows a network that detects the presence of two components and its performance on simulated data (Figures 3B,C). Note that the weights of the input units and the threshold of the output units must be tuned to ensure that the output unit will be active *only* under the presence of *both* compounds. Specifically, the influence of the inputs must be capped to ensure that neither could activate the output unit on its own. For linear summation of n components, each with a maximal input of 1, setting the threshold in the range [*n*-1, 1] will satisfy the condition. However, as the number of components is increased, the ratio *(n*-*1)*/*n* approaches unity, and small random fluctuations can lead to activation of the output unit even without the presence of all components. Non-linear, synergistic, input summation (Silver, 2010), as has been demonstrated for olfactory cortex neurons (Davison and Ehlers, 2011), can at least partially resolve the problem of accidental activation by only a subset of the inputs. In a related scenario, a given biological trait could involve the presence of some compounds, combined with the *absence* of others. An example is the inhibitory effect of heterospecific cues on flight in moths (Lelito et al., 2008). This computation can be realized by an output unit that receives both excitatory and inhibitory inputs that reflect the levels of each of these compounds (Figures 3D–F). To enforce the requirement for the absence of a specific compound, its inhibitory effect must be large enough to “veto” activation of the output unit. As in the previous example, this is more difficult when the output unit integrates many excitatory inputs. More generally, it may be required to detect whether levels of each of multiple compounds fall within particular ranges. This can be achieved by a network (Figures 3G–I) that includes an output unit receiving inputs from two range-detection networks such as those shown in Figure 2. Note that because the decision rules embodied by the networks in Figures 3A–G refer to the *sum* of compounds, they do not impose a specific condition about the level of the individual compounds. Graphically, this results in decision boundaries with diagonal lines in the 2D decision space (Figures 3C,F,I). This is distinct from a situation in which *each* of the compounds is above a certain value, or within a given range. The latter condition can be achieved if the modules associated with each individual compound impose a threshold (or range), as shown in Figures 3J–L for the case of detection of two components.

### Multi-compound codes involving relationships

The scenarios above involved traits that were associated with levels of multiple compounds, but not by explicit relationships among them. Yet, in both vertebrates and invertebrates, specific *relationships* among compounds can be highly informative and often constitute the important message. In ants, for instance, the relative *proportions* of multiple cuticular hydrocarbons provide the basis for colony recognition (Martin et al., 2008), and in mice, ratios of distinct major urinary proteins provide information about the stimulus donor (Kaur et al., 2014). Although such analog codes present challenges for readout, they allow enhanced coding capacity as compared to codes defined by the presence or absence of individual components. An example for a postulated binary code is the use of major urinary proteins to convey individuality (Cheetham et al., 2007). More generally, analyses of mouse (Zhang et al., 2007), and rat (Zhang and Zhang, 2011) urinary profiles have shown that the relative abundance (rather than the presence or absence) of particular components is the best indicator of relatedness among different strains. Another fundamental reason for the importance of relationships involves the temporal and/or spatial aspects of the stimulus source. The concentration of a single volatile component decays with the distance from its source. Likewise, the concentration will change with time at any distance. For a soluble component, evaporation of either the solvent or the compound will lead to concentration changes. In a multiple molecule mix, if rates of diffusion/dispersion and evaporation are similar for all compounds, then evaluation of their ratios (at any point in time and space) provides a better estimate of stimulus identity than any component in isolation. Indeed, it has been shown that rats can discriminate binary odor mixtures based on the ratios of the components (Uchida and Mainen, 2007). Furthermore, the rats can generalize their decision rules to other mixtures with identical component ratios, but different total concentrations. On the other hand, if two or more compounds have different (and known) dispersal or evaporation rates, as well as known concentrations at the original stimulus source, then their concentration ratios can provide information about the distance of the stimulus source or the time since its deposition. Various species can discriminate fresh urine from old urine, and this could be achieved by comparing levels of multiple components with different volatility (Rich and Hurst, 1999).

**Figure 4:**
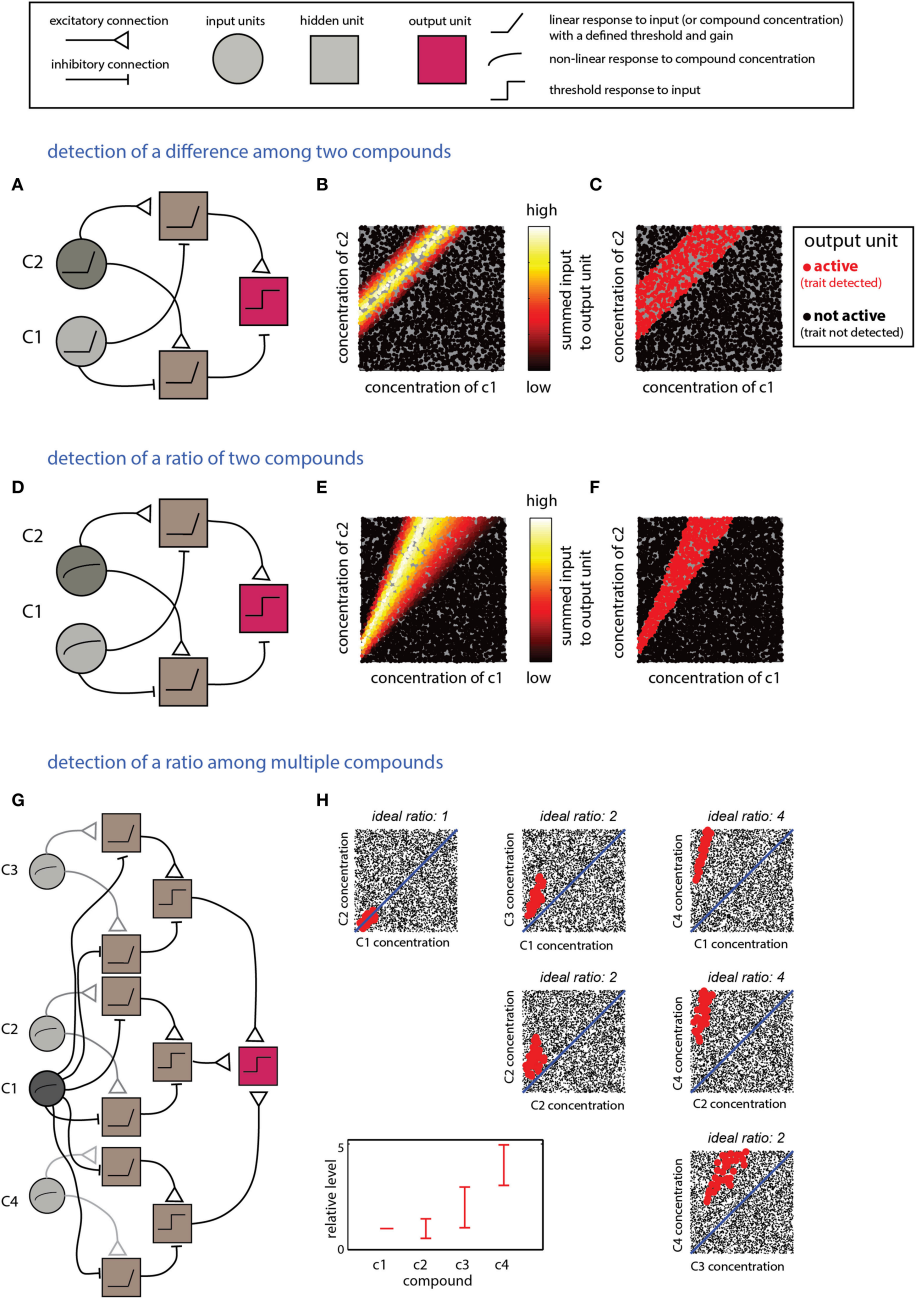
**Detection of explicit relationships between compounds. (A–C)** Detection of a difference range. The output unit will respond if the difference between two compounds is within a specific range. **(A)** The network comprises two input units which respond linearly (with a threshold) to the two components. Each of the two second-layer units respond to the difference between the two compounds but with a different offset. The output unit responds to the difference between the second layer units. **(B)** Inputs into the output unit as a function of the levels of c1 and c2. **(C)** Activity in the output unit after thresholding. The threshold sets the width of the difference range around the ideal difference. **(D–F)** Detection of a ratio range. **(D)** The network is very similar to that shown in **(A)** for detection of a difference, except that the input units respond linearly to the log of the compound concentration. **(E)** Inputs to the output unit in **(D)** as a function of c1 and c2. **(F)** Output of the unit in **(D)** after thresholding the inputs in **(E)**. **(F,G)** Detection of mixtures defined by specific proportions among components. **(G)** A network that detects a mixture of four components (c1–c4) with specific ratio ranges. The network comprises three modules with the same layout as that in **(D)**. Each of the modules compares one of the compounds (c2–c4) to c1. The outputs of these modules are integrated by the output unit that performs a threshold operation on the summed outputs of the three modules. **(H)** Representation of the output of the unit. Each mixture is defined within a 4D space which is shown here by all pairwise projections. The relevant proportions relative to c1 (i.e., those associated with a trait) are indicated in the bottom left panel. Mixtures classified by the network as associated with the trait are shown in red (others are shown in blacks). Thus, each mixture is represented in each of the panels. Legend at top explains network elements.

The scenarios described in this section require computations that detect relationships between multiple compounds. Integration of excitatory and inhibitory inputs from units reflecting levels of individual compounds can be used to derive information about differences or the ratios between them. Linear input units are suitable for calculating differences, whereas logarithmic responses (or their approximations) are naturally suitable for calculating ratios (Uchida and Mainen, 2007). The networks in Figure 4 implement detection of difference- (Figures 4A–C) or ratio-ranges (Figures 4D–F) between two compounds. The two networks differ only in the response function of the input units. The more general situation, involving relationships among more than two compounds, can be addressed by combining several networks such as those in Figures 4A,D. One possible network, and its respective decision boundary for a specific ratio range among four compounds, is shown in Figures 4G,H. Note that this network utilizes one “anchor” component (c1) to which all the others are referenced. An elegant solution to a similar pattern recognition problem, using temporal coding, has been raised by Hopfield (1995) and subsequently elaborated by Brody and Hopfield (2003). Logically, the networks shown in Figure 4G and that suggested by Brody and Hopfield (2003) are similar, but whereas the Hopfield network detects a pattern defined by a fixed ratio, the network in Figure 4D allows each compound to vary within a certain ratio *range*.

### Context dependent trait-compound relationships

**Figure 5:**
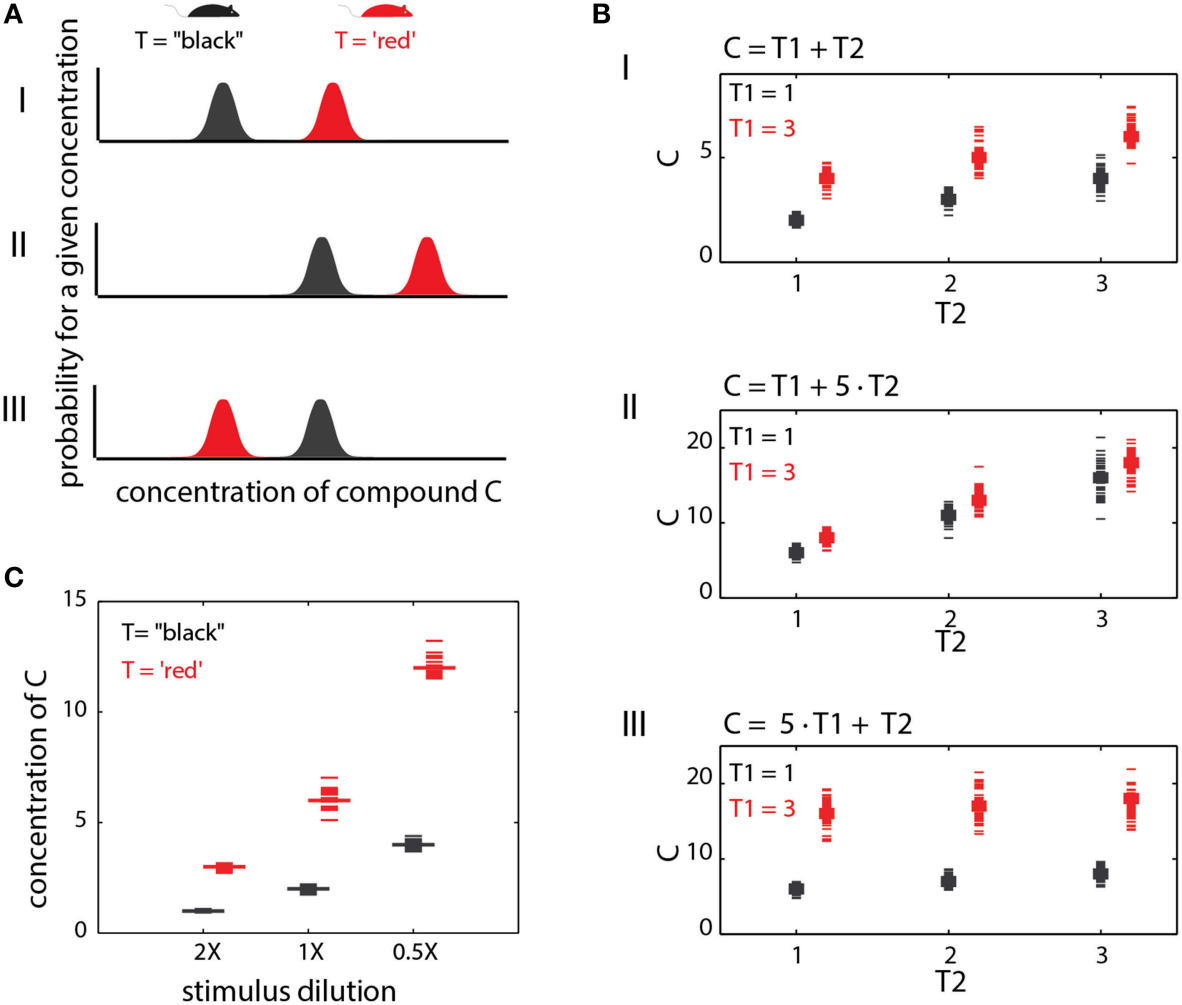
**Context effects. (A)** Context dependent changes in compound distributions. This example illustrates a single cue with a different distribution under two trait (T) values. **(AI)** shows a baseline condition where the two distributions are non-overlapping. A simple threshold can be set to distinguish the trait values (T = “black” vs. T = “red”) based on the level of **(C)**. In **(AII)**, both distributions are shifted. The threshold used in **(AI)** will no longer yield reliable discrimination among the two traits. **(AIII)** the relationships between compound levels and trait values are reversed. Here, the red trait is associated with lower values of **(C)**. **(B)**. Interaction between two different traits in determining compound levels. The first trait (T1) can take one of two values (1 or 3), while the second can take the values 1, 2, or 3. In **(BI)**, both traits exert an equal influence on the levels of **(C)**, which is simply their sum. In **(BII)**, the influence of T2 is dominant, while in **(BIII)**, T1 dominates. **(C)** Effect of dilution on specific compound levels. The example illustrates how different dilutions can confound trait identification.

In the preceding sections, only individual traits were considered, but in fact, the levels of any chemical compound may depend on *several* traits. Consequently, the relationship between any one trait and chemical profiles will be context dependent. This notion is supported by experimental evidence. For instance, comparative analysis of urinary components across sex and strain (Zhang et al., 2007; Zhang and Zhang, 2011) or across strains and reproductive states (Schwende et al., 1984), revealed that many of the individual components are modulated by both of these traits. Consistent with this observation, a search for chemical markers of diet, maturation, stress and diurnal rhythm (Schaefer et al., 2010) revealed a large overlap between reliable markers for each of these factors, indicating that many individual markers are modulated by multiple factors (or traits). This idea is illustrated graphically in Figure 5AI for a trait reflected by levels of a single compound (as in Figure 2). The presence of the “red” trait, and its absence (black) are associated with distinct probability distributions of the corresponding compound. If the distributions do not overlap, then perfect discrimination can be achieved by a simple network such as shown in Figure 2A. Overlapping distributions present a different complication which is not considered here.

If a compound's level depends on multiple traits, then identification of any particular trait from that compound, requires consideration of all others. Figure 5A illustrates this idea with two hypothetical scenarios. In the first (Figure 5AII), there is a general shift of compound level distributions as compared to the reference condition (Figure 5AI). Here, a different decision threshold is required for correctly detecting the “red” trait. In the more exotic case, shown in Figure 5AIII, the *direction* of change as a function of the trait also depends on other traits. Importantly, these particular scenarios are (qualitatively) evident from actual measurements of urinary volatiles for different strain and reproductive-state combinations (Schwende et al., 1984). Consequently, a network such as shown in Figure 2A simply cannot reliably detect the “red” trait across all the conditions shown in Figure 5A. To illustrate the effect of multiple traits more formally, consider a particular compound i, whose concentration levels C_i_ are determined by a linear combination of two traits, T_1_ and T_2_, i.e., C_i_(T_1_, T_2_) = g_1_•T_1_+ g_2_•T_2_. The gain factors g_1_ and g_2_ determine the influence of each trait on the compound's concentration. The traits are not binary, but can assume one of several numerical values. In Figure 5BI, g_1_ and g_2_ are equal, so both traits exert the same influence. In Figure 5BII, the second trait dominates (g_1_ < g_2_), while in Figure 5BIII, the first trait is dominant. In Figure 5BI, neither trait can be determined without knowing the other. In Figures 5BII,III, the dominant trait can be determined without knowledge of the other, but not vice-versa.

Sometimes, the context can be set by physical, rather than physiological factors. One example is stimulus source dilution. Figure 5C shows the effects of 2X and 0.5X dilutions of the original stimulus source on compound levels. As in the other examples above, it is not possible to discriminate among trait values without knowledge of the stimulus dilution. Here too, the confounding effect of stimulus dilution depends on how strongly different trait values affect compound distributions. For example, if distinct trait values exert a 100-fold change on a given compound, then the confounding effect of a 2-fold dilution will be minor. On the other hand, if different trait values induce a 2-fold change, then the confounding effect of the same dilution will be critical. Another circumstance where context plays a role is during social investigation, where animals typically sample multiple body regions to obtain information about each other (Johnston, 2003; Luo et al., 2003; Kimoto et al., 2005; delBarco-Trillo et al., 2009; Liberles, 2014). For example, determining an individual's sex from urinary cues could involve very different rules as compared to salivary cues. Finally, note that context dependence is not limited to single compound codes as even the *relationships* among compounds may change under different physiological contexts.

### Accounting for context dependence using simple networks

**Figure 6:**
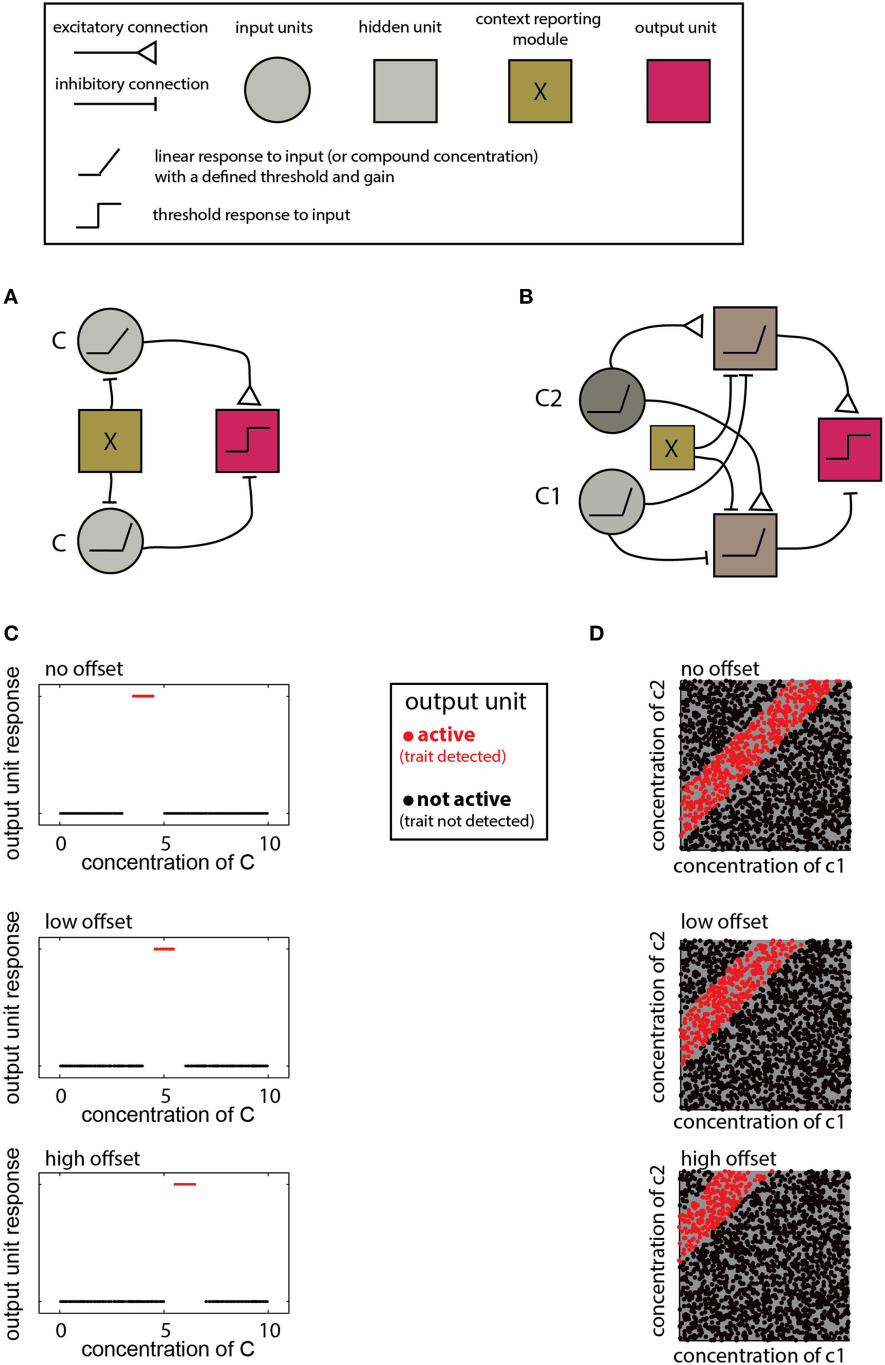
**Implementation of context dependent rules. (A)** Introduction of a simple bias term (via the context detecting module X) can shift the range detected by a single-compound range-detecting network. **(B)** Similarly, a bias term can change the range associated with a two-component-difference detecting network. **(C)** Different offsets into the network in **(A)** change the detection range of **(C)**. **(D)** Different offsets into the network shown in **(B)** alter the detected difference range. In both **(A,B)**, insertion of different offsets into each of the two units that feed into the output unit can change the upper and lower bounds of the range independently. Legend at top explains network elements.

The networks described above are designed to detect a certain *type* of relationship between compound compositions and a trait. The specific decision criteria for each network are determined by the properties of individual units and their connections. Adjustment of these parameters allows learning of novel distributions and accounting for contextual effects. For example, detecting different ranges as a function of context, as required by the scenarios in Figure 5B, calls for a simple modification of a range-detecting network. Such a network is shown in Figure 6A where the context is taken into account by introducing an offset to the input units, thereby altering the decision-range (Figure 6B). Another example for flexible decision rules is shown in Figure 6C, where the offset determines the difference-range for which the network is sensitive.

The contexts accommodated by the networks in Figure 6 are simple and applicable under very limited conditions. Namely, the context must be defined by a trait that can be coded numerically and which modifies the compound distribution in a simple (e.g., monotonic), manner. In other cases, the context may call for altering the lower and/or the upper limit of a decision range, or even reverse its direction (e.g., Figure 5AIII). For multi-component distributions, the context may influence the distribution of only a subset of components. This can be accounted for by modifying a subset of elements or connections within the network. For example, in the ratio detecting network shown in Figure 4G, specific elements can be tuned to change the required proportion of particular compounds. Note that although the context may be reflected by the level of some other compound, context detection itself may be complicated and could involve complex chemical features, or even sensory information from other modalities. In such cases, it may be easiest to simply integrate information from distinct networks, each of which is designed for a particular context.

## Discussion

To efficiently map external information to appropriate behavioral outcomes (Purves, 2010) sensory systems must be tuned to the informative statistical features of the environment (Barlow, 2001). Like early vision processing stages that detect regions with high contrast, or lines with specific orientations (Hubel, 1988), early chemosensory processing is likely designed to extract predictable statistical motifs associated with natural stimuli. This implies that understanding chemosensory circuits requires understanding the statistical structure of chemosensory signals. The complexity of chemosensory scenes implies a corresponding complexity of the neuronal circuits detecting them. Below, I discuss whether chemosensory stimuli must really be complex and attempt to map the abstract elements of the networks shown above to actual components of the olfactory system.

### Do chemosensory signals really have to be so complex?

Throughout this manuscript, it was assumed that chemosensory information is conveyed by relationships among multiple compounds. It may be argued that this is an unjustified complication, and that chemical communication only requires detection of individual components. Analyses of natural stimuli reveal that some components are uniquely associated with one sex (Lin et al., 2005; Chamero et al., 2007; Zhang et al., 2007; Haga et al., 2010; Roberts et al., 2010) but such studies compare sex-specific expression patterns across a limited number of genetic backgrounds. Indeed, even when a small number of strains is considered, at least some cues show strain dependence. For example, some murine signals may be associated exclusively with one sex, but only for some strains (Kimoto et al., 2007; Ferrero et al., 2013). Although these findings are based on inbred strains, similar, though perhaps smaller effects, are also likely across individuals in wild populations.

Physiologically, responses to social stimuli have been studied mainly in the context of the vomeronasal system (VNS). Often, neuronal responses at the first brain relay of the VNS, the accessory olfactory bulb (AOB), are sex and strain-specific (Luo et al., 2003; Ben-Shaul et al., 2010; Tolokh et al., 2013). While some neurons, both in the vomeronasal organ (He et al., 2008), and the AOB (Ben-Shaul et al., 2010) do reveal consistent sex-specific responses across multiple strains, the number of tested strains is limited. Basing chemical communication on individual compounds, especially for traits that are subtler than sex, does not exploit the immense combinatorial coding capacity of the olfactory system.

Extraction of traits, rather than identifying specific individuals, is important because they are key to guiding specific behaviors. Because in chemosensation, the relevant quantities are compound levels, and because compound levels are generally influenced by multiple factors, decoding any one trait must take into account knowledge of others. Comparison of levels of various urinary cues in juvenile and sexually mature female mice, across three strains of mice (Schwende et al., 1984), revealed that relative levels of virtually all reported compounds were affected by both the strain *and* the reproductive state of the female. These scenarios closely resemble those shown in Figure 5A, and thus provide a direct illustration that decoding a female's reproductive status from chemical cues requires prior knowledge about her genetic background. In addition to these physiological considerations, physical aspects such as stimulus source dilution will also modulate (and complicate) chemosensory scenes. Much of the variability induced by such physical factors can be resolved by considering relationships among multiple components. While much remains unknown about the statistical nature of chemosensory scenes, these lines of evidence suggest that extraction of information from them, requires consideration of multiple components and their relationships.

### Mapping computations to actual elements of the olfactory system

The computational steps described above used the metaphor of networks, but they are not intended to reflect real neuronal elements and synaptic interactions. Implementing some of the processing ascribed here to single units could require multiple neurons (Linster and Cleland, 2009) while on the other hand, some computations assigned to distinct units may be implemented by single neurons. Indeed, individual complex neurons, such as mitral/tufted cells (MTCs), can implement multiple processing stages using distinct cellular compartments (Silver, 2010). Despite these caveats, assuming that the networks reflect real computations, it should be possible to map them to specific elements within the olfactory system. Although there are many similarities between invertebrates and vertebrates (Kaupp, 2010), the discussion below focuses on vertebrate chemosensory systems.

A given odorant can activate multiple receptor types (with different affinity), and likewise, a single receptor type can be activated by multiple odorants (Malnic et al., 1999). This is true for both single molecules and multi-component mixtures. As a consequence, at increasing concentrations, any given odorant will activate a larger set of glomeruli (e.g., Spors and Grinvald, 2002) confounding stimulus intensity and quality. Various solutions have been suggested for maintaining responses to a given odor-stimulus similar across a range of concentrations (Cleland et al., 2011). Here, I simplify and assume that each compound in a multi-compound mixture activates one type of sensory neuron (defined by its receptor), corresponding to a single input unit in the networks shown above.

The input units in all networks exhibit a linear or a logarithmic response to individual compounds. Obtaining such response profiles by single sensory neurons is not trivial and integration across several receptors with different dynamic ranges is likely required to achieve a consistent response across a broad range (Cleland et al., 2011). In the current context, note that different computations call for different stimulus response relationships. For instance, detection of differences is facilitated by responses that scale linearly with the concentration (e.g., Figure 4A), whereas ratio detection is better accomplished by responses that are linear with the logarithm of the concentration (e.g., Figure 4D). Other networks involved units that are sensitive to compound levels within a certain range (Figure 2). While more complicated, these non-monotonic responses still involve only a single compound, and do not require comparison of activity across distinct channels. Accordingly, all computations involving a single compound could in principle be implemented by intra-glomerular circuits. The considerable complexity of olfactory bulb (OB) circuits suggests several potential ways to implement these computations (Shepherd et al., 2004; Nagayama et al., 2014). For example, intra-glomerular inhibition onto MTCs via periglomerular cells (Nagayama et al., 2014) could form a basis for shaping the slope and range of the relationship between odor concentration and response magnitude. Indeed, glomerular circuits are implicated in various processes including response linearization and decorrelation (Cleland et al., 2011; Banerjee et al., 2015) and contrast enhancement (Cleland and Sethupathy, 2006). For a thorough discussion of glomerular interactions, see (Cleland, 2014). Overall, then, it seems reasonable to map activity related to specific individual components to apical dendrites of MTCs, which reflect the result of glomerular level computations. Indeed, response functions of MTCs show various patterns of dependency on stimulus concentration, including non-monotonic responses that are consistent with detection of a certain compound range (Harrison and Scott, 1986; Meredith, 1986; Wellis et al., 1989). Furthermore, at least in the AOB, it was shown that MTCs can display responses that scale linearly with the logarithm of stimulus concentration (Arnson and Holy, 2013).

The next computational step requires integration of information from multiple channels. While inter-glomerular connections exist, they are generally believed to relay global information (Cleland, 2014; Banerjee et al., 2015), and are thus less suitable to mediate specific interactions among individual channels. The same is likely true for paravalbumin-positive interneurons in the external plexiform layer (Kato et al., 2013; Miyamichi et al., 2013). In contrast, interactions within the external plexiform layer allow specific interactions between MTCs and granule cells (Nagayama et al., 2014). Granule cells receive excitatory inputs from MTCs, and provide inhibition unto the same, or to other MTCs via dendro-dendritic synapses (Shepherd et al., 2004), whose efficacy depends on the distance of the synapse from the MTC soma (Gilra and Bhalla, 2015). This type of connectivity provides an enormous combinatorial capacity for specific interactions. The existence of multiple sister MTCs, (i.e., with apical dendrites sampling from the same individual glomerulus), could allow each to interact with a unique set of non-sister MTCs (Gilra and Bhalla, 2015). Despite similarity in firing rates in response to single odors, sister MTs show distinct timing with respect to the breathing cycle phase (Dhawale et al., 2010). This difference between sister MTCs could be due to distinct inputs that each receives and which are likely to shape the timing with respect to the sniff cycle (Soucy et al., 2009). Thus, sister MTCs are expected to reveal even more dramatic differences in their responses to *combinations* of multiple components than to differences in responses to individual compounds.

A serious problem with assigning across-channel integrative function to MTC-granule cell circuits is that many of the computations require *summation* across distinct channels, and it is not obvious how this can be achieved with the inhibitory MTC-granule cell network. Therefore, it is tempting to discount a central role for OB circuits in coding combinations and attribute all such integrative processing to the cortex, where there is extensive potential for both summative and subtractive processing (Poo and Isaacson, 2011; Bekkers and Suzuki, 2013). This fits with the notion that OB circuits implement basic and preliminary computations like concentration invariance, stimulus decorrelation, or linearization (Miyamichi et al., 2013; Adam et al., 2014; Cleland, 2014; Uchida et al., 2014), while representations of complete patterns and objects are generated in the olfactory cortex (Sosulski et al., 2011; Chapuis and Wilson, 2012). However, the OB may nevertheless play a role in integrative and summative processing. For example, if a MTC is constantly suppressed by some granule cells, reduced activity in those granule cells will lead to MTC disinhibition. Thus, MTC responses might effectively summate by mutually disinhibiting each other. In this context, one study concluded that responses of individual MTCs to combined stimuli are linear sums of responses to individual components (Gupta et al., 2015), while another study found a few cases where the responses to stimulus combinations were qualitatively different from those of the elemental stimuli (Giraudet et al., 2002). Explicitly testing summation by MTCs requires measurement of responses across a fuller extent of the high dimensional stimulus spaces considered here (e.g., 2D space shown in Figures 3B,E,H,K and Figures 4B,E).

The seemingly limited magnitude of cross-talk (inhibitory, and particularly excitatory) between MTCs, at least when measured as firing rate changes, raises the possibility that temporal coding could play a role in these interactions. The exact timing of MTC responses, particularly within the sniff cycle, carries important information about odor identity (Cury and Uchida, 2010; Shusterman et al., 2011; Smear et al., 2011). As noted above, sister MTCs show distinct timing with respect to the breathing cycle phase (Dhawale et al., 2010) possibly due to selective lateral inputs from specific glomerular channels (Soucy et al., 2009). Interestingly, MTC phase-responses are approximated by negative and positive contributions from distinct glomerular channels with opposing effects on the response within the sniff cycle (Soucy et al., 2009). Olfactory cortex neurons are tuned to particular stimulus combinations (Lei et al., 2006; Davison and Ehlers, 2011), in a particular temporal order (Haddad et al., 2013; Sanders et al., 2014). Thus, cortical integration of MTC responses could depend on the temporal *alignment* of MTC responses within the sniff cycle, to allow efficient summation within a constrained time window (Uchida et al., 2014). Consistent with this idea, in MTCs, mixtures of two components that elicit distinct phase responses, elicit intermediate phase responses (Khan et al., 2008). Experimentally testing this hypothesis requires evaluation not only of the magnitude, but also of the temporal features of responses to multi-component stimuli.

The next brain stage, the olfactory cortex, seems ideal for implementing both summative (Lei et al., 2006; Apicella et al., 2010; Davison and Ehlers, 2011; Poo and Isaacson, 2011) and subtractive (Suzuki and Bekkers, 2012; Sturgill and Isaacson, 2015) processing via feedforward and recurrent connectivity. Due to the complexity of cortical circuits, however, it is even harder to speculate how specific computations can be mapped onto particular cortical elements. This connectivity provides a rich substrate for implementing multiple stages of processing, including those called for by the networks described here. The notion of cortical integration using the fine temporal aspects of MTCs firing lies at the heart of the model of Hopfield and Brody (Brody and Hopfield, 2003). In broad terms, the model can be thought of as a realization of the more abstract networks shown here (i.e., Figure 4G). However, while the Hopfield and Brody model defines a pattern as a combination of odorants at some particular relative proportion, the networks here allow for more general patterns defined by certain *ranges* of stimulus levels or ratios among them, as well as the absence of other stimuli.

### Distinctions between the main and vomeronasal olfactory system

An important point that was overlooked thus far is the distinction between the main and the vomeronasal olfactory systems. Although the role of both in social behaviors is well established (Keller et al., 2009; Tirindelli et al., 2009; Stowers and Logan, 2010; Korzan et al., 2013; Beny and Kimchi, 2014; Liberles, 2014), a large body of studies specifically implicates the VNS in social behaviors. Some of the functional differences between the two systems are directly relevant here. For example, unlike the main OB, apical dendrites of AOB MTCs can sample information from multiple glomerular channels (Takami and Graziadei, 1991; Wagner et al., 2006; Larriva-Sahd, 2008). This provides an obvious opportunity for summing inputs from distinct channels and raises the idea that the different connectivity between the systems reflects differences in the statistics of the stimuli that they evolved to detect. Presently, it is not known which computations are realized by this connectivity. While there is evidence for synergistic processing across channels (Ben-Shaul et al., 2010), it does not appear to be the only mode of processing in the AOB (Meeks et al., 2010). Another important difference between the main olfactory and vomeronasal systems is that neuronal responses in the latter are not locked to the sniff cycle, but rather to the non-periodic activation of the vomeronasal organ (Meredith, 1994; Luo et al., 2003; Ben-Shaul et al., 2010). This difference may call for distinct modes of integration by downstream neurons of the VNS. Recent studies have provided important insights about the physiology of the medial amygdala (MeA) (Martinez-Marcos, 2009), a key region receiving inputs from AOB MTCs (Bian et al., 2008; Bergan et al., 2014; Hong et al., 2014; Keshavarzi et al., 2014). Further studies of these regions should reveal the computations realized by them.

### Summary and outlook

A comprehensive understanding of how social information is communicated and detected involves several lines of research (Figure 1). Logically, the first stage involves identification of traits that can actually be detect by particular organisms. The second involves characterization of the chemical compound distributions associated with each trait. Third is the investigation of the relationships between neuronal activity and particular patterns of chemical cues. This requires measurement of neuronal activity, across multiple populations, to high-dimensional stimulus spaces. Such knowledge, combined with a better understanding of the connectivity should reveal how the computations discussed here are implemented by the nervous system. This last effort will greatly benefit from realistic computational models to focus physiological hypotheses and experiments.

### Conflict of interest statement

The author declares that the research was conducted in the absence of any commercial or financial relationships that could be construed as a potential conflict of interest.
